# Physics-Informed Monotonic Conformer for Remaining Useful Life Prediction of Hydraulic Systems

**DOI:** 10.3390/s26072178

**Published:** 2026-04-01

**Authors:** Xiansong He, Chen Zhang, Jinyuan Wang, Xiaoli Zhao, Jianyong Yao, Chuanjie Lu, Xiaowei Yang

**Affiliations:** 1 School of Mechanical Engineering, Nanjing University of Science and Technology, Nanjing 210094, China; brownsong@njust.edu.cn (X.H.); wangjny@njust.edu.cn (J.W.); yaojianyong@njust.edu.cn (J.Y.); 2 Department of Armored Vehicle Technology Campus of Urumqi, Engineering University of PAP, Urumqi 830049, China; qdzc2017@163.com; 3 Dezhou Precion Machine Tool Co., Ltd., Shandong Precion Group, Dezhou 253799, China; luchuanjie6929@163.com

**Keywords:** prognostics health management, hydraulic systems, physics-informed machine learning, conformer, monotonicity constraint

## Abstract

Reliable heavy machinery requires accurate health assessments of its hydraulic systems. Existing data-driven models often fail to track long-term degradation trends while concurrently ignoring the physical laws governing wear. This oversight produces predictions that contradict the natural irreversible progression of equipment faults. This study introduces the Physics-Informed Monotonic Conformer to address this specific problem. The proposed model combines convolutional inductive biases with global self-attention to merge multi-scale spatiotemporal features. We also implement a monotonicity loss function to enforce physical degradation constraints. This step grounds the purely data-driven network in actual physical realities. Testing on an electrohydrostatic actuator dataset shows the new method surpasses current baseline models. The regularization mechanism also significantly improves physical consistency, yielding a high Spearman rank correlation. The resulting health indicators offer the numerical precision and physical reliability necessary for safety-critical aerospace deployment.

## 1. Introduction

Hydraulic power transmission systems are indispensable in contemporary industrial applications, including aerospace [1], construction engineering [2], and manufacturing [3]. The reliability and efficiency of these systems hinge upon the structural integrity and health of critical components, namely pumps, servo valves, and hydraulic cylinders. Nevertheless, subjected to rigorous environments involving high pressure and dynamic loading profiles, these elements are prone to inevitable performance deterioration. Unscheduled failures not only result in extensive downtime but also incur severe safety hazards [4]. Therefore, the implementation of reliable PHM strategies [5] is imperative for early fault detection and mitigation. Moreover, the development of quantitative metrics for accurate wear assessment constitutes the core of predictive maintenance and Remaining Useful Life (RUL) estimation.

Current research extensively leverages deep learning [6,7] paradigms to distil latent features from raw sensory data [8]. Specifically, Convolutional Neural Networks [9] are frequently employed to identify local patterns within vibration or pressure signals, while recurrent architectures [10] such as LSTM [11] and GRU [12] demonstrate efficacy in capturing temporal dependencies. Despite these advancements, conventional models encounter distinct constraints in complex hydraulic system monitoring. The finite receptive field inherent in convolutional layers limits the capacity to capture long-term degradation trends. Simultaneously, recurrent networks incur prohibitive computational costs and are susceptible to gradient instability when processing ultra-long sequences. Although the Transformer architecture [13,14] addresses sequence-length bottlenecks through its global self-attention mechanism, standard Transformer models remain insufficient for early-stage fault detection in hydraulic systems [15,16]. Specifically, these models lack directional sensitivity toward critical features such as rapid pulses and micro-fluctuations, thereby struggling to accurately characterize subtle degradation signatures.

Reliable condition monitoring for complex hydraulic systems cannot be achieved by merely optimizing network architectures. There remains a significant gap between purely data-driven models and the actual physical mechanisms of these systems. In practice, high-fidelity datasets that cover the entire degradation process are extremely scarce [17,18]. Most existing models are trained on limited samples with discrete labels, which often leads to poor generalization and a lack of physical consistency [19]. Because standard training algorithms focus solely on minimizing numerical errors, they tend to overlook the underlying laws of mechanical wear and irreversible degradation [20]. Therefore, a new diagnostic framework is needed to integrate physical constraints with data learning, ensuring that predictions strictly follow the irreversible nature of system degradation [21,22].

To mitigate this, recent studies have pioneered the integration of physical constraints, such as monotonicity and physical degradation priors, into deep learning frameworks to ensure prognostic reliability [23,24,25]. While these existing physics-guided models have significantly advanced the field, adapting them to the complex, dual-scale nature of hydraulic systems—which exhibit both rapid high-frequency transient impulses and long-term aging trends—remains challenging.

Building upon these foundational physics-informed approaches, this study proposes a Physics-Informed Monotonic Conformer (PIMC) architecture designed to reconcile data-driven insights with physical regularities. The proposed architecture integrates local and global representation mechanisms tailored to specific hydraulic signal characteristics at the algorithmic level. Specifically, convolutional modules are embedded within the Transformer encoder structure to enhance the resolution of high-frequency components and facilitate the extraction of incipient, weak anomaly features. Concurrently, the self-attention mechanism is tasked with modeling global degradation trajectories to capture long-term dependencies and systemic evolution trends. This hybrid configuration effectively unifies the detection of transient responses with the modeling of progressive aging, thereby establishing a physically consistent feature space for the health assessment of hydraulic systems.

The main contributions of this paper are summarized as follows:

1. A hydraulic-specific conformer architecture is proposed. This design utilizes convolutional bias to help the standard transformer identify subtle, local fault signatures.
2. A physics-informed loss mechanism is introduced. This translates the principle of irreversible degradation into differentiable constraints, preventing the model from generating physically implausible predictions.
3. Extensive testing on hydraulic data demonstrates the effectiveness of the proposed method.

## 2. Related Work

### 2.1. Self-Attention Mechanism and Global Dependency Modeling

**Figure 1:**
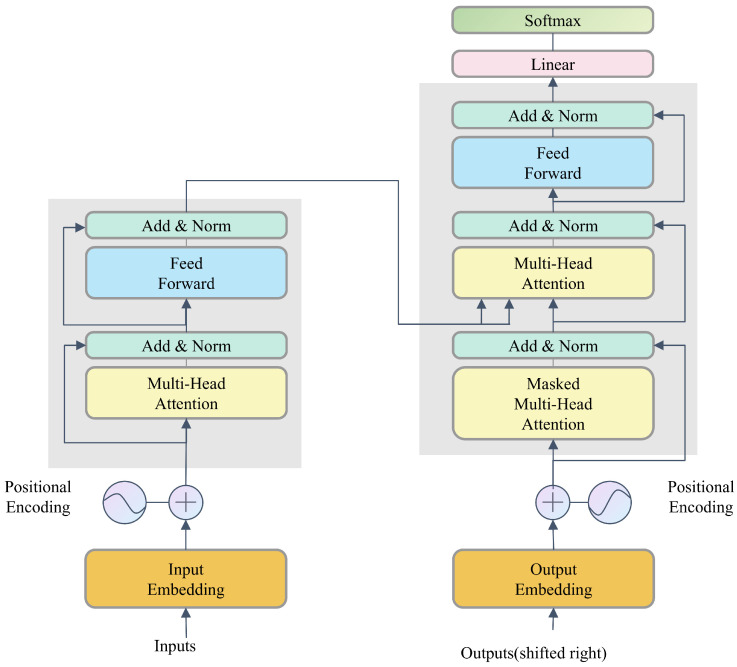
The transformer architecture.

Self-attention mechanisms have fundamentally transformed our approach to sequence modeling as shown in Figure 1. Unlike traditional Recurrent Neural Networks, which are constrained by sequential processing and decaying long-term memory, self-attention facilitates direct correlation across all sequence positions. This architecture enables the mapping of global dependencies without the need for sequential recursion. Consequently, the model can simultaneously characterize both local and remote interactions, remaining unaffected by the temporal span between individual data points.

We define an input feature sequence $H\in R^{T\times d}$. The variable *T* represents the total sequence length while *d* specifies the exact feature dimension. The algorithm linearly projects this raw input into three distinct latent subspaces known as the Query Q, the Key K, and the Value V. This projection step relies entirely on learnable weight matrices $W^{Q},W^{K},W^{V}\in R^{d\times d_{k}}$:

(1) $$Q=HW^{Q},\;K=HW^{K},\;V=HW^{V}$$

Within the self-attention mechanism, scaled dot-product operations are employed to compute the final attention weights. The process begins with the multiplication of the query and key matrices to generate alignment scores, which represent the correlations between different positions in the sequence. To prevent excessive gradients and ensure training stability in high-dimensional spaces, these scores are normalized by the square root of the key vector dimension. Subsequently, a Softmax function transforms these scaled scores into a probability distribution, providing a precise quantification of the attention weights across the input sequence.

(2) $$Attention\left(Q,K,V\right)=softmax\left(\frac{QK^{T}}{\sqrt{d_{k}}}\right)V$$

The standard Transformer architecture treats input sequences as unordered sets, lacking explicit mechanisms to model positional sequences and spatial locality. Unlike CNN, which possess inherent translation invariance, the Transformer fails to sufficiently capture subtle spatio-temporal correlations within a sequence. In the context of hydraulic condition monitoring, this structural limitation hinders the detection of early-stage degradation. During initial failure phases, hydraulic components typically exhibit localized pressure anomalies. Although these signals have small amplitudes, they are critical for characterizing degradation trends. Global self-attention tends to average out such localized disturbances during computation, effectively masking fine-grained patterns associated with wear evolution. Consequently, the standard Transformer remains inadequate for the precise identification and characterization of early degradation in hydraulic systems.

### 2.2. Principles of the Conformer Architecture

Gulati et al. [26] introduced the conformer architecture to address the local feature extraction limitations native to transformers. This network design integrates the spatial translation invariance of convolutional layers with the global context awareness of self-attention mechanisms. The resulting hybrid framework demonstrates proficiency in analyzing complex sequential data.

The conformer modifies the standard transformer block layout by adopting a macaron inspired structural arrangement. For any given input sequence $H_{l-1}$ passing into layer *l*, the tensor flows through two half step feed forward networks. These twin networks physically bracket a central multi-head self-attention block alongside a dedicated convolution module. The mathematical sequence executes as follows:

(3) $$\begin{array}{cc} \tilde{H} & =H_{l-1}+\frac{1}{2}\mathrm{FFN}\left(\mathrm{LN}\left(H_{l-1}\right)\right)\\ H^{\prime } & =\tilde{H}+\mathrm{MHSA}\left(\mathrm{LN}\left(\tilde{H}\right)\right)\\ H^{\prime \prime } & =H^{\prime }+\mathrm{ConvModule}\left(\mathrm{LN}\left(H^{\prime }\right)\right)\\ H_{l} & =\mathrm{LN}\left(H^{\prime \prime }+\frac{1}{2}\mathrm{FFN}\left(\mathrm{LN}\left(H^{\prime \prime }\right)\right)\right) \end{array}$$

The variable LN represents standard layer normalization, which is interleaved with the self-attention mechanisms, enabling the network to maintain a dynamic balance between local feature refinement and global information aggregation during the forward pass. To address the inherent limitations of standard Transformers in local temporal modeling, the embedded convolutional module is designed to introduce an explicit local inductive bias. This architectural modification enhances the model’s capacity to characterize short-term dynamic patterns within hydraulic signals. The convolutional module performs a sequence of transformations on the intermediate feature tensors, which can be mathematically formulated as:

(4) $$\mathrm{ConvModule}\left(X\right)={\mathrm{PW}}_{2}\left(\sigma_{\mathrm{swish}}\left(\mathrm{BN}\left(\mathrm{DW}\left(\mathrm{GLU}\left({\mathrm{PW}}_{1}\left(X\right)\right)\right)\right)\right)\right)$$

The variables PW and DW represent pointwise and depthwise convolutions. The term BN indicates batch normalization while $\sigma_{\mathrm{swish}}$ specifies the swish activation function. This configuration separates standard convolution operations into distinct depthwise and pointwise phases. The separation significantly reduces computational overhead. The network simultaneously maintains its capacity to capture highly localized temporal contexts.

This dual stream mechanism builds a theoretical foundation for analyzing mechanical hydraulic signals. It enables the architecture to extract high-frequency transient impulses and low-frequency degradation trends concurrently.

## 3. Proposed Method

### 3.1. Overall Framework

**Figure 2:**
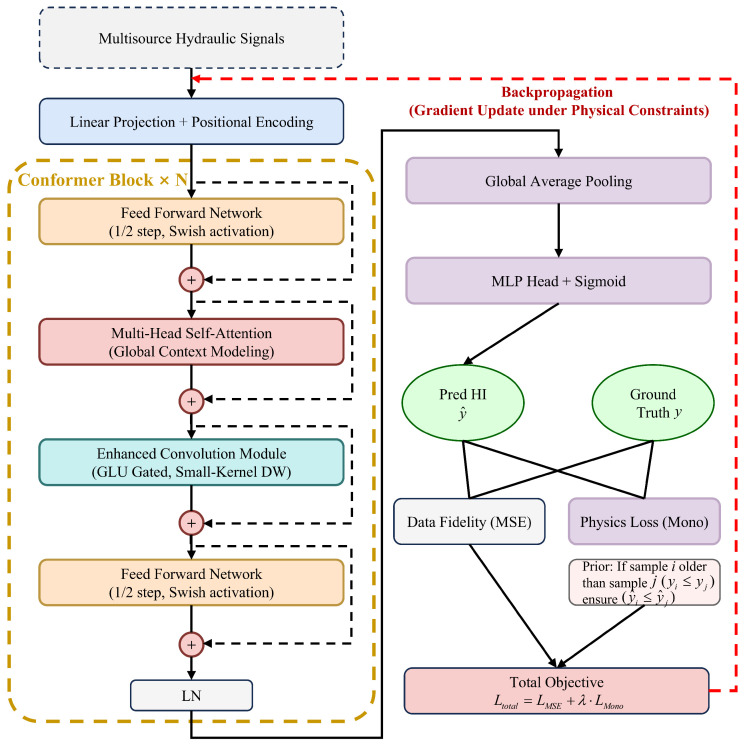
The proposed framework.

To overcome the aforementioned limitations of purely data-driven models, we engineered the PIMC as shown in Figure 2. This hierarchical structure translates raw heterogeneous sensor data directly into continuous health scores. The complete diagnostic process operates across three distinct stages.

An upgraded conformer encoder serves as the foundation during the first phase. Integrated convolutional layers capture rapid transient impulses while native self-attention blocks simultaneously map the long term degradation curve. A global pooling layer then compresses the high dimensional spatiotemporal tensors. A non-linear regression head projects these vectors directly into a continuous health space for quantitative tracking. We introduce a custom monotonicity loss function during the training phase. This mathematical formulation strictly enforces the irreversible nature of mechanical wear. The constraint aligns all final network predictions with actual physical degradation paths.

### 3.2. Hydraulic Conformer Design

Speech recognition applications originally popularized the standard conformer network. Applying this baseline model directly to a hydraulic system proves ineffective. Fluid mechanics generate distinctly different physical signal signatures. We modified several core structures to optimize the network specifically for mechanical condition monitoring. The specific architectural configuration and layer parameters of the proposed Hydraulic Conformer are summarized in Table 1. The following sections detail these architectural adjustments.

Step-by-Step Architectural Explanation and Feature Fusion Strategy: To clarify the data flow and feature fusion, the network pipeline is summarized as follows:

1. Signal Embedding: Raw multivariable sensors are projected into a latent space and fused with learnable positional encodings to preserve temporal sequence order.
2. Macaron-Style Blocks: The core extraction utilizes three identical Conformer blocks. Each block employs a “macaron-like” residual connection scheme, sandwiching the multi-head self-attention (MHSA) and convolutional modules between two half-step Feed-Forward Networks (FFNs) for stable gradient flow.
3. Local-Global Fusion: Within each block, the MHSA module first captures global, long-range degradation dependencies. Subsequently, the custom Convolutional Module extracts high-frequency localized impulses. Notably, a Gated Linear Unit (GLU) is applied before the large-kernel depthwise convolution, acting as an adaptive fusion gate to filter irrelevant noise.
4. HI Generation: The temporal features are compressed via global average pooling and mapped into a continuous HI bounded between [0, 1] through a Sigmoid-activated regressor.

#### 3.2.1. Signal Embedding and Positional Encoding

Hydraulic condition monitoring requires the analysis of continuous multivariate time series streams. A linear projection layer extracts the raw sensor matrix $X\in R^{T\times C}$ and maps it directly into a high dimensional latent space $d_{\mathrm{model}}$. Self-attention operations lack inherent awareness of sequence order. We superimpose sinusoidal positional encodings directly onto the projected embeddings. This procedure ensures strict temporal ordering and resolves the inherent permutation invariance. The initial hidden state is calculated as follows:

(5) $$H_{0}=\mathrm{Linear}\left(X\right)+P$$

#### 3.2.2. Enhanced Convolution Module

Degrading piston pumps and sticking valve spools generate pressure pulsations and flow shocks lasting fractions of a millisecond. Standard architectures frequently overlook these rapid physical transients. We modified the baseline convolution module by incorporating advanced gating mechanisms alongside depthwise separable convolutions. The data tensor undergoes the following computational sequence:

(6) $$H_{\mathrm{conv}}=\mathrm{BN}\left(\sigma_{\mathrm{swish}}\left(\mathrm{DW}\left(\mathrm{GLU}\left({\mathrm{PW}}_{1}\left(H\right)\right)\right)\right)\right)$$

The module decomposes standard convolution into channel projection and temporal feature extraction, reducing computational complexity while preserving localized temporal context. This enables the network to resolve subtle time-varying patterns in hydraulic signals with higher resolution. The resulting convolution attention architecture facilitates complementarity between local and global representations, establishing a robust framework for early degradation monitoring. By hierarchically associating high-frequency transients with low-frequency trends, the model integrates short-term disturbances and long-term evolution to achieve precise degradation characterization.

#### 3.2.3. Global Pooling and Regression Head

For continuous health tracking, high-dimensional features are compressed into a scalar health indicator. The architecture substitutes the standard decoder with a Global Average Pooling (GAP) layer and a regression head. GAP aggregates temporal features into a global state vector, which a Multi-Layer Perceptron (MLP) then maps to the health indicator. To ensure physical consistency and interpretability, a sinusoidal activation function bounds the output within [0,1]. This topology effectively balances model complexity with monitoring accuracy, providing a unified framework for long-term degradation assessment.

(7) $$\hat{y}=\sigma \left(\mathrm{MLP}\left(\mathrm{GAP}(H_{L})\right)\right)$$

### 3.3. Physics-Informed Loss Function

To eliminate the physically implausible predictions inherent in standard MSE optimization, We developed a specialized composite loss function to integrate strict data fidelity with physical constraints and eliminate these monotonic violations.

#### 3.3.1. Data Fidelity Term

The network must still learn the baseline observational data accurately. We utilize standard mean squared error to calculate the absolute deviation between the predicted health indicator $\hat{y}$ and the true ground truth label *y*:

(8) $$L_{\mathrm{MSE}}=\frac{1}{N}\sum_{i=1}^{N}{\left({\hat{y}}_{i}-y_{i}\right)}^{2}$$

#### 3.3.2. Physics-Consistency Term

Wear fatigue and aging represent an irreversible physical process inside hydraulic components. To mathematically formalize this prior knowledge, we ground our approach in the classical Archard’s wear law. This physical law establishes that the wear volume *V* is proportional to the sliding distance *L* and the normal load *W*, formulated as:

(9) $$V=K\frac{WL}{H}$$

where *K* is the wear coefficient and *H* is the material hardness. Under continuous operation of a hydraulic system, the cumulative sliding distance L(t) is a strictly monotonically increasing function of time *t*. Consequently, the time derivative of system degradation (cumulative wear) is inherently non-negative: dV(t)/dt≥0.

The system’s health indicator (HI) serves as an inverse function of this cumulative wear. Therefore, the true health indicator y(t) must follow a strict monotonic non-increasing trajectory. For any given time steps $t_{i}<t_{j}$, the physical inequality $y_{i}\geq y_{j}$ must hold.

We translate this fundamental physical inequality directly into a differentiable pairwise penalty. We evaluate any given training sample pair *i* and *j*. If the true label identifies sample *i* (occurring earlier in time) as healthier than sample *j*, the network must mathematically ensure ${\hat{y}}_{i}\geq {\hat{y}}_{j}$. The algorithm applies a specific penalty whenever the predicted trend contradicts this law of irreversible physical degradation:

(10) $$L_{\mathrm{mono}}=\frac{1}{M}\sum_{i,j}\mathrm{ReLU}\left(-\mathrm{sgn}\left(y_{i}-y_{j}\right)\;\cdot \;\left({\hat{y}}_{i}-{\hat{y}}_{j}\right)\right)\;\cdot \;I(|y_{i}-y_{j}\left|>\epsilon \right)$$

The sign operator extracts the true direction of physical degradation. The rectified linear unit triggers the mathematical penalty only when the predicted trend contradicts physical laws. An indicator function applies a predefined threshold ϵ to filter out random label noise occurring between adjacent health states. This dedicated term operates as a physical regularizer. It constrains the optimization manifold to physically plausible degradation paths.

Computationally, this formulation calculates the pairwise differences between predicted states and applies a directed penalty term via the ReLU activation only when the prediction direction violates the physical ground truth. The indicator function I incorporates a noise threshold ϵ to prevent over-penalization from minor measurement fluctuations.

#### 3.3.3. Total Objective Function

The complete optimization objective linearly combines the raw data fitting error and the strict physical consistency penalty:

(11) $$L_{\mathrm{total}}=L_{\mathrm{MSE}}+\lambda \;\cdot \;L_{\mathrm{mono}}$$

It is important to note that the monotonicity loss is applied as a soft regularizer during the backpropagation process, rather than a hard architectural constraint. A dedicated hyperparameter λ directly controls the strength of this physical soft regularizer. Tuning this coefficient balances the mathematical data fitting precision against the network’s adherence to physical rationality.

## 4. Experimental Verification

### 4.1. Dataset Description and Preprocessing

**Figure 3:**
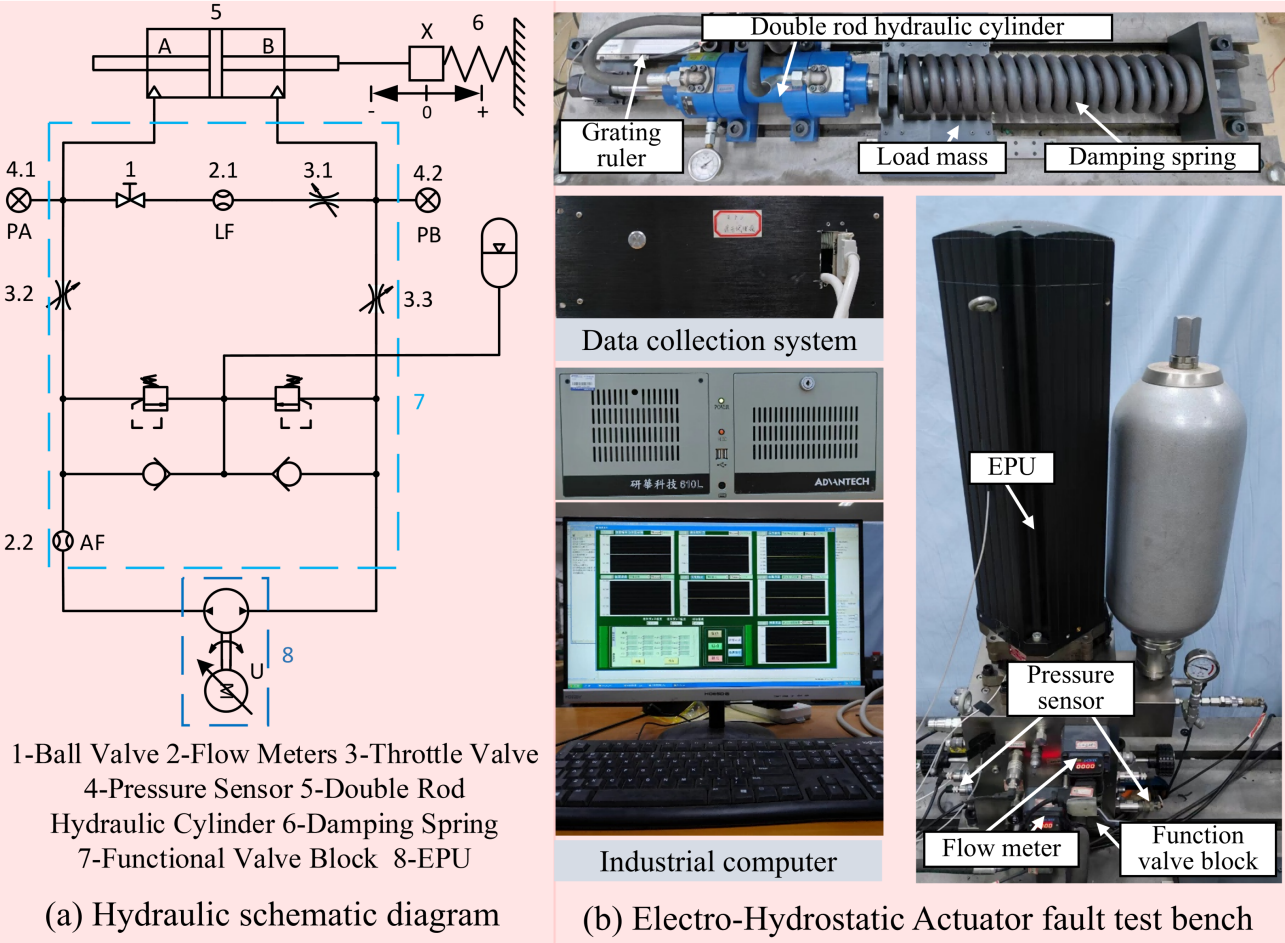
The EHA test bench.

To validate the proposed method, we conduct experiments on an EHA test bench as shown in Figure 3. As a typical pump-controlled hydraulic system, the EHA is highly representative of modern integrated hydraulic equipment. Therefore, it serves as an ideal physical platform for validating our condition monitoring and prognostic framework.

#### 4.1.1. EHA Test Bench

The physical test environment combines a main electrohydrostatic pump unit with a core hydraulic circuit and a mechanical damping spring assembly. An industrial control computer and a dedicated data acquisition module manage the complete hardware setup. The master computer transmits precise control signals to run the system servo motor. This motor drives the central hydraulic pump and pushes the connected cylinder through its full operational stroke. Table 2 lists the hardware parameters for these main components. To further clarify the dynamic and physical operating environment of the EHA dataset, the main physical and structural parameters of the experimental platform are detailed in Table 3. The dataset is collected in-house from our physical EHA test bench under controlled operating conditions. The system continuously operates against a constant load mass of 13.5 kg and a compression spring (stiffness: 245,000 N/m), driven by a 0.2 Hz, 20 mm amplitude sinusoidal position command.

#### 4.1.2. Data Acquisition and Labeling Strategy

The hydraulic rig runs under a continuous sine wave for position control. We fixed the oscillation amplitude at 20 mm with a 0.2 Hz frequency to represent typical mechanical duty cycles. The hardware captures all sensor data at a steady 200 Hz sampling rate throughout the test. Our system logs four primary variables during these experiments. These data streams include fluid pressure from both cylinder chambers alongside the mechanical piston position and the raw servo command signal.

Feeding the entire raw recording directly into the network proves inefficient. We slice the continuous time series into overlapping windows instead. This segmentation step creates a much larger dataset and enables the architecture to learn the specific temporal patterns within the hydraulic signals. The window size spans exactly 1000 time steps with an overlap ratio of 0.4. We apply standard Z score normalization individually across each sensor signal channel to eliminate dimensional inconsistencies.

We configured the system with 12 discrete fault levels based on the magnitude of internal leakage measured in liters per minute per bar to simulate different degrees of degradation. The process maps these discrete fault levels to continuous health indicator labels ranging from 1 to 0. The value 1 represents a healthy state while 0 represents complete failure. Table 4 details the exact definition of these fault levels and their corresponding leakage rates.

### 4.2. Experimental Setup

#### 4.2.1. Baseline Models

We constructed four baseline configurations based on two widely used deep learning architectures to evaluate the performance of the proposed method comprehensively. This approach decouples the specific contributions of the network architecture from the physics-informed loss function.

The bidirectional long–short-term memory network represents classic recurrent architectures. It captures temporal dependencies but suffers limitations from serial computation. We evaluate two specific variants. The standard MSE variant utilizes only the mean squared error loss during training. The physics-enhanced variant incorporates our proposed monotonicity loss to test its adaptability.

The transformer model represents self-attention-based architectures. It excels in global context modeling but lacks local inductive bias. We evaluate a standard MSE variant trained solely on fitting error alongside a physics-enhanced variant trained with the monotonicity constraint.

We tuned the hyperparameters across all models to control the number of trainable parameters uniformly near 300,000 with a 10% margin. This uniform capacity ensures a fair comparison and focuses the evaluation strictly on performance differences resulting from architectural design.

#### 4.2.2. Evaluation Metrics

We evaluate the proposed framework across two distinct dimensions. The testing protocol measures pure mathematical fitting precision alongside actual physical degradation consistency.

Three standard mathematical metrics quantify the numerical difference between the network prediction $\hat{y}$ and the true ground truth label *y*. We calculate the mean squared error, the root mean squared error, and the mean absolute error for every test sequence. Minimizing these error values confirms the baseline predictive accuracy of the diagnostic algorithm. The definitions of these metrics operate as follows:

(12) $$MSE=\frac{1}{N}\sum_{i=1}^{N}{\left({\hat{y}}_{i}-y_{i}\right)}^{2}$$

(13) $$RMSE=\sqrt{\frac{1}{N}\sum_{i=1}^{N}{\left({\hat{y}}_{i}-y_{i}\right)}^{2}}$$

(14) $$MAE=\frac{1}{N}\sum_{i=1}^{N}\left|{\hat{y}}_{i}-y_{i}\right|$$

The variable *N* represents the total number of evaluated samples.

Hydraulic component wear follows a strictly irreversible physical path. We deploy the Spearman rank correlation coefficient ρ to evaluate the monotonicity of all network predictions. This statistical tool evaluates the sequential ranking alignment between the forecasted health values and the true degradation stages. A correlation score approaching one indicates the predicted trajectory safely obeys the physical reality of continuous decline. Maximizing this metric reduces the risk of spurious recovery anomalies during live operation.

#### 4.2.3. Implementation Details

We document the computational environment and model complexity to ensure experimental reproducibility and confirm aerospace deployment feasibility.

The complete experimental suite runs on a dedicated workstation featuring an Intel Core i9-13900K processor and an NVIDIA GeForce RTX 4060Ti graphics card. The software stack utilizes Python 3.12.9 alongside the PyTorch 2.6.0 framework. An AdamW optimizer drives the network training phase. We define the initial learning rate at $1\times 10^{-3}$ and manage its decay through a cosine annealing schedule. The algorithm processes training batches of 64 samples and completes optimization after 100 epochs. Empirical testing fixed the physics constraint balancing coefficient λ at 2.0 for the proposed network architecture. A shuffled test dataset verifies the final model robustness against random data variance.

We analyze the detailed estimation outcomes across all five model configurations to confirm prediction stability and distribution characteristics throughout every physical degradation stage.

### 4.3. Analysis of Main Comparative Results

**Figure 4:**
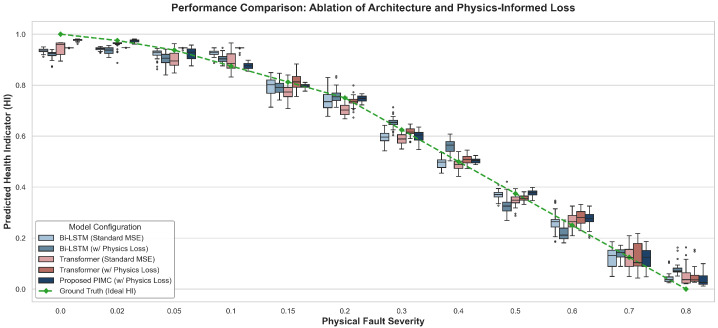
Boxplot comparison of predicted health indicators across different physical fault severities for all evaluated model configurations.

As shown in Figure 4, evaluating the prediction distributions reveals distinct behavioral differences across all tested algorithms. Baseline models relying exclusively on standard data fidelity loss produce large interquartile ranges. These networks generate overlapping prediction bounds between adjacent physical fault severities. The transformer architecture is highly sensitive to minor local signal fluctuations. This inherent oversensitivity results in scattered prediction outliers that deviate from the actual degradation path.

Applying the custom physics-informed penalty shrinks the prediction variance across both baseline structures. The monotonicity constraint acts as a powerful mathematical regularizer. Our proposed physics-informed monotonic conformer framework achieves a compact prediction distribution at every physical fault stage. The network clusters its estimated health indicators directly along the ideal ground truth trajectory. We observed no reverse trend anomalies during testing. Merging local global feature fusion with physical consistency regularization successfully restricts the optimization solution space. The complete algorithm delivers the robust and credible prognostic results required for active industrial deployment.

Table 5 details the quantitative performance metrics for our proposed framework against the four baseline configurations using the independent test set. We decoupled the specific contributions of the network architecture from the applied loss strategy to explicitly demonstrate the superior performance of our combined method.

To further highlight the architectural advantages of the proposed method, it is crucial to examine the model’s performance during the incipient fault stage. During this early phase, degradation features are exceedingly subtle, highly localized, and frequently masked by normal operational noise. Transformer baselines exhibit scattered prediction outliers and wider variance. This occurs because global self-attention mechanisms tend to average out localized, small-amplitude disturbances. In contrast, the proposed PIMC model maintains a significantly tighter prediction distribution that closely aligns with the ground truth. This superior early-stage sensitivity directly validates the integration of the enhanced convolutional module. By introducing a strong local inductive bias, the convolution operations successfully capture the micro-fluctuations and high-frequency transient impulses characteristic of early incipient wear, thereby preventing delayed fault detection.

#### 4.3.1. Visual Analysis of Experimental Results

To explicitly elucidate the mechanism by which the proposed framework enforces physical plausibility, both the temporal prognostic trajectories and the high-dimensional latent feature space are visualized.

**Figure 5:**
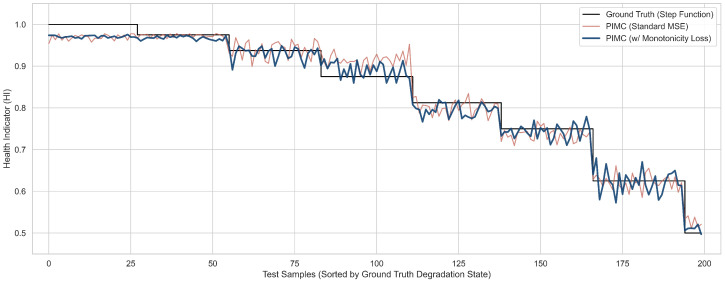
Visual evidence of prognostic trajectories. The model trained with standard MSE exhibits unphysical fluctuations, whereas the proposed PIMC framework with the monotonicity loss correctly predicts a physically plausible, non-increasing degradation path.

The evolution of the predicted Health Indicator (HI) is presented in Figure 5. Standard mean squared error optimization minimizes point-wise numerical distance but remains completely agnostic to the irreversible physics of mechanical wear. Consequently, the purely data-driven model overfits to localized sensor noise. This results in highly jagged predictions across the degradation plateaus, which correspond to physically impossible spontaneous health recoveries. The integration of the monotonicity regularizer systematically suppresses these unphysical anomalies.

**Figure 6:**
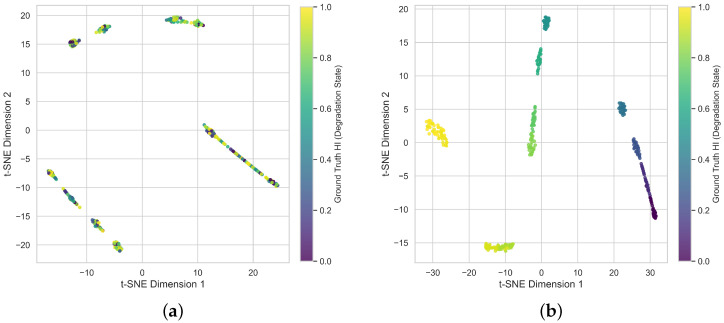
Impact of physical regularization on the latent feature manifold. (**a**) Features with Standard MSE. (**b**) Features with Monotonicity Constraint. The monotonicity constraint forces the entangled features to reorganize into a strictly sequential and physically interpretable degradation manifold.

The fundamental driver of this behavioral shift is observed in the latent feature embeddings, visualized via t-SNE in Figure 6. Figure 6a demonstrates that without physical regularization, the latent representations extracted by the network are fragmented. Features from distinct degradation stages exhibit localized entanglement and lack a cohesive temporal progression. The network maps individual inputs to outputs without understanding the underlying wear continuum. In contrast, Figure 6b shows that under the monotonicity constraint, the network reorganizes the latent space into a distinct and sequentially ordered manifold. The feature clusters exhibit a continuous, unidirectional transition from the healthy state to complete failure, perfectly mirroring the physical degradation timeline.

This structural reorganization directly accounts for the substantial improvement in the Spearman rank correlation. Standard optimization permits arbitrary rank permutations due to local noise fitting, which inherently degrades the correlation metric. Mapping the temporal features onto a physically constrained, descending manifold ensures that the final predicted severities strictly preserve their true chronological ordering. This intrinsic rank preservation eliminates random numerical fluctuations and mathematically guarantees the exceptional Spearman correlation score achieved by the proposed framework.

#### 4.3.2. Numerical Accuracy Analysis

The numerical fitting results demonstrate the value of our custom loss function alongside the conformer architecture design. The physics-informed penalty significantly influences the base transformer architecture. Upgrading from standard mean squared error optimization to our custom physical loss reduces the transformer root mean square error from 0.0398 to 0.0369. This represents a 7.3% performance improvement. The structural monotonicity constraint functions as an effective regularizer. It steers the core self-attention mechanism away from random high-frequency noise fitting and aligns the network with the actual degradation manifold.

Testing the best-performing physics-regularized transformer against our complete framework reveals an additional root mean square error reduction from 0.0369 to 0.0265. These performance improvements confirm that our combined network design extracts features much more precisely than standalone recurrent or transformer networks. Fusing local convolutional inductive biases directly with global attention mechanisms drives this diagnostic accuracy.

#### 4.3.3. Physical Consistency Analysis

In safety-critical aerospace scenarios, standard error metrics like MSE are insufficient. A model might achieve low average error but still predict physically impossible “health recoveries” due to local fluctuations. For an EHA system, such non-monotonic anomalies could cause maintenance crews to dangerously delay critical component replacements. Therefore, the Spearman rank correlation coefficient (ρ) is essential. It strictly quantifies adherence to irreversible mechanical wear, ensuring that prognostic trajectories are monotonically non-increasing and thus safe for real-world deployment.

Algorithms relying entirely on standard mean squared error optimization demonstrate flawed physical consistency. Table 5 shows the baseline recurrent network achieving a correlation score near 0.9683. The network occasionally violates physical reality through impossible spontaneous recovery events due to this lower monotonicity alignment. Integrating the physical loss formulation repairs this specific vulnerability across every tested architecture.

Our complete framework produces the highest recorded consistency score of 0.9941. This high correlation value indicates that the predicted trajectory adheres closely to the actual physical degradation path. The newly designed architecture successfully mitigates the occurrence of spurious recovery anomalies. These structural constraints successfully bound the entire predictive solution space. The generated health indicators maintain extreme numerical accuracy while strictly obeying fundamental physical laws. This dual reliability provides the diagnostic confidence required for live deployment inside safety-critical aerospace platforms.

## 5. Conclusions

To address the tendency of purely data-driven models to diverge from physical laws, this study proposes the PIMC for hydraulic health assessment. By integrating convolutional inductive biases into a transformer encoder, PIMC concurrently captures high-frequency transient impulses and long-term degradation, reducing the RMSE to 0.0265. Furthermore, a custom monotonicity loss function acts as a physical regularizer to penalize impossible “health recovery” anomalies. This ensures strict adherence to irreversible wear principles, achieving a high Spearman rank correlation of 0.9941.

Despite these improvements, certain limitations exist. Currently, validation is confined to an EHA test bench, necessitating future generalization testing across diverse hydraulic systems. Additionally, PIMC was not benchmarked against other physics-informed paradigms like PINNs. Since PINNs rely on explicitly defined PDEs—which are highly challenging to analytically formulate from complex EHA signals—direct comparison was structurally difficult. Future work will focus on establishing a cross-paradigm evaluation framework and compressing the network for edge computing deployments.

## Figures and Tables

**Table 1:** Architectural configuration of the proposed Hydraulic Conformer.

Module/Layer	Key Components & Operations	Input Dim.	Output Dim.
Input	Raw multisource hydraulic signals	-	B×1000×4
1. Embedding	Linear Projection + Positional Encoding	B×1000×4	B×1000×64
2. Conformer Block	2.1 Half-Step FFN 1 (+Residual)	B×1000×64	B×1000×64
(Repeated ×3)	2.2 Multi-Head Attention: 4 heads (+Residual)		
	2.3 Conv Module: GLU Gating → Depthwise Conv1d (k = 31) → Pointwise Conv (+Residual)		
	2.4 Half-Step FFN 2 (+Residual)		
3. Aggregation	Global Adaptive Average Pooling	B×1000×64	B×64
4. Regressor	MLP (Swish) + Sigmoid Activation	B×64	B×1

**Table 2:** Specification of the main components of the EHA test bench.

Name	Specification
EPU	Moog SEPU019ADN1H0C
Pressure sensor	MEAS-US175-C00002-200BG
A/D Converter card	Advantech PCI-1716
Grating ruler	Heidenhain LC485
D/A Converter card	Advantech PCI-1723
Counter card	Heidenhain IK-220
Industrial computer	Advantech IPC-610

**Table 3:** Main physical parameters of the EHA test bench.

Parameter	Value
Safety Valve Set Pressure	16 MPa
Hydraulic Pump Displacement	0.019 mL/rev
Rated Rotational Speed	3000 rev/min
Hydraulic Cylinder Stroke	0.1 m
Effective Piston Area	1.527 × 10^−3^ m^2^
Initial Volume of Both Chambers (at mid-position)	7.635 × 10^−5^ m^3^
Accumulator Pressure	0.6 MPa
Compression Spring Stiffness	245,000 N/m
Oil Elastic Modulus	700 MPa
Load Mass	13.5 kg

**Table 4:** Definition of fault levels based on internal leakage rates.

Health Indicators	Leakage Rate (L/min/bar)	Description
1	0.00	Baseline Health
0.975	0.02	Incipient Fault
0.935	0.05	↓
0.875	0.10	↓
0.8125	0.15	↓
0.75	0.20	Progressive Degradation
0.625	0.30	↓
0.5	0.40	↓
0.375	0.50	↓
0.25	0.60	Severe Fault
0.125	0.70	↓
0	0.80	Near Failure

Note: The arrows (↓) indicate the continuous progression and increasing severity of the specific fault category as the leakage rate increases.

**Table 5:** Comprehensive Ablation Study and Quantitative Performance Metrics on the Test Set.

Architecture	Loss	Params (k)	FLOPs (M)	RMSE	MAE	R^2^	CRA	ρ
Bi-LSTM	MSE	313.5	608.3	0.0553	0.0463	0.9723	0.9229	0.9705
	mono	313.5	608.3	0.0411	0.0349	0.9848	0.9419	0.9821
CNN	MSE	377.6	620.7	0.0676	0.0559	0.9588	0.9068	0.9846
	mono	377.6	620.7	0.0644	0.0466	0.9625	0.9224	0.9886
Transformer	MSE	398.6	529.5	0.0398	0.0312	0.9857	0.9481	0.9774
	mono	398.6	529.5	0.0369	0.0295	0.9877	0.9509	0.9856
**Proposed**	MSE	308.7	385.9	0.0269	0.0212	0.9934	0.9647	0.9900
	**mono**	308.7	385.9	**0.0265**	**0.0211**	**0.9937**	**0.9648**	**0.9941**

Note: Bold values indicate the best performance results among all evaluated models.

## Data Availability

The data presented in this study are available on request from the corresponding author due to data are derived from the National Science and Technology Major Project of China, which is still under execution and subject to confidentiality and proprietary restrictions.
